# hPSC-derived organoids: models of human development and disease

**DOI:** 10.1007/s00109-020-01969-w

**Published:** 2020-08-28

**Authors:** Tristan Frum, Jason R. Spence

**Affiliations:** 1 Department of Internal Medicine, Gastroenterology, University of Michigan Medical School, Ann Arbor, MI USA; 2 Department of Cell and Developmental Biology, University of Michigan Medical School, Ann Arbor, MI USA; 3 Department of Biomedical Engineering, University of Michigan College of Engineering, Ann Arbor, MI USA

**Keywords:** Stem cells, Development, Organoids, Human, Disease modeling, Cell signaling

## Abstract

Organoids derived from human pluripotent stem cells (hPSCs) have emerged as important models for investigating human-specific aspects of development and disease. Here we discuss hPSC-derived organoids through the lens of development—highlighting how stages of human development align with the development of hPSC-derived organoids in the tissue culture dish. Using hPSC-derived lung and intestinal organoids as examples, we discuss the value and application of such systems for understanding human biology, as well as strategies for enhancing organoid complexity and maturity.

## Introduction

During development, a small aggregate of pluripotent cells in the embryo are guided through a series of cell fate decisions to generate the incredible diversity of cell types required for human life. Along the way, multiple specialized cell types organize into complex 3D structures to build organs, which perform essential physiological tasks such as respiration in the lungs, nutrient absorption in the gut, and filtering of blood in the kidneys. For centuries, scientists have worked to understand the process of development and to identify the cellular and molecular cues that guide cells to generate a particular organ. Now, scientists are applying this knowledge to recapitulate development in a cell culture dish, guiding cells to organize into complex 3D models of human organ-like form and function, structures known as organoids.

Organoids permit scientific investigation of human development, physiology, and disease at a scale and level of precision not previously possible. Traditionally, scientists have relied on a combination of animal models and human 2D cell culture models to investigate human biology. While these approaches have led to innumerable important discoveries, animal models and 2D cell culture models are not without their shortcomings. Animal models are complex, making it difficult to discern cause and effect for many experiments, provide limiting amounts of tissue for analysis, and by their nature are imperfect models of human physiology. Traditional human 2D cell culture models on the other hand are often too simple, containing a single cell type attached to the culture dish as a monolayer, or floating as single cells in suspension culture. Moreover, 2D cell culture models are typically derived from cancer or induced to a cancer-like state using viral oncogenes, permitting indefinite propagation of these models in vitro, but also leading to genomic instability that makes these models become less like the cell types they are derived from[1–3]. In these examples, human cells grown in 2D cell culture also fail to recapitulate the cell-cell interactions and 3D architecture critical to organ physiology and function. Organoids, by contrast, can be derived or generated from healthy human cells, contain many of the cell types present in an organ, maintain a stable genotype/phenotype, and retain aspects of organ architecture, physiology and function [4–7].

Methods to establish primary organoids from many different human organs have been published, including the intestine [8], pancreas [9, 10], stomach [11, 12], lung [13–17], and liver [4]. In general, conditions that establish and maintain organoids induce a highly proliferative state in organ resident stem cells by recapitulating cues from the stem cell niche that promote stem cell self-renewal and differentiation during normal tissue homeostasis or in response to injury in vivo. The stem cell niche varies in composition across different organs and stem cell compartments and is composed of physical and chemical cues, such as the extracellular matrix and growth factor signaling molecules, respectively. Thus, the act of establishing conditions to maintain organoids is in itself informative for understanding how the stem cell niche regulates the behavior of stem cells in normal and pathological states across many different organ systems.

Organoids can be established from healthy or diseased patient tissue and have numerous basic and translational applications including disease modeling, personalized medicine, and for the development of therapeutics. For instance, using material derived from patients organoid models of cystic fibrosis [18–20], polycystic kidney disease [21, 22], Hermansky-Pudlak syndrome [23], and even complex neurological disorders such as Rett syndrome [24] have been established. Similarly, healthy organoids can be used to model disease onset [25–27], which is of particular interest as the early stage of disease is often the most challenging to observe in vivo and when therapeutic intervention is likely to be most effective. Importantly, organoid models can be established from any patient, and therefore have the potential to capture patient-specific aspects of any disease and guide the personalization of therapeutics. To this end, several groups have harnessed the proliferative capacity of organoid models to develop high-throughput screening methodologies against a variety of diseases, laying the groundwork for the development of therapies that address the patient specific aspects of disease [5, 18, 19, 28, 29].

While patient-derived organoids are commonly employed for in vitro modelling of human organ physiology, limited access to some patients and genetic diversity within the human population make patient-derived organoids less suitable for some studies. This has enhanced interest in the establishment of methods to generate organoids from human pluripotent stem cells (hPSCs), which are renewable, widely available, and are maintained under highly standardized culture conditions [30, 31]. In general, protocols to generate organoids from hPSCs attempt to mimic human development and organogenesis in a step-wise manner. In contrast to patient-derived organoids, which more closely resemble adult tissue, hPSC-derived cells and organoids often are more similar to immature or fetal tissue undergoing development [32–37]. In addition, hPSC-derived organoids can possess non-desired cell types [33, 38, 39], likely due to imperfect differentiation/maintenance conditions. To this end, researchers working to improve hPSC-derived organoid models have relied of the utilization of fetal tissue to provide a road-map to improve hPSC-derived organoid models, and as an essential in vivo reference to benchmark hPSC-derived organoids [17, 33, 40–45].

Here we will provide an overview of the generation of hPSC-derived organoids, highlighting the contribution of developmental biology to understanding the physical and molecular cues that guide the development of cells in the culture dish. We will focus on directed differentiation of hPSCs towards definitive endoderm, en route to making hPSC-derived lung organoids (HLOs) and hPSC-derived intestinal organoids (HIOs). We discuss strategies to drive organoid maturation and to circumvent current limitations to organoid complexity. Finally, we discuss current and potential applications well suited to hPSC-derived organoids.

### From pluripotency to germ layer specification

To generate organoids, researchers harness the developmental potential of hPSCs, which have the capacity to differentiate into any cell type in the human body—a property known as pluripotency. hPSCs are established by isolating pluripotent embryonic stem cells from early human embryos [46], or by inducing differentiated cells to reacquire pluripotency [47]. hPSCs can be expanded in the laboratory under conditions that maintain pluripotency, or alternatively guided through a series of cell fate decisions to generate desired cell types, an approach termed directed differentiation. Directed differentiation can be thought of as a tree of cell fate decisions. Cells proceed through these cell fate decisions in a step-by-step fashion, with each step further committing a cell towards the desired cell type, while restricting the potential to generate alternative cell types. A series of these decisions culminating in the formation of a fully differentiated cell type is referred to as a cell lineage or collectively as a lineage tree. Importantly, comparison of single-cell RNA sequencing from human fetal tissue and from hPSC-derived organoids and their intermediates demonstrates that the structure of lineage trees appears to be broadly consistent whether cells are undergoing development in vivo or in a culture dish [17, 33, 40–45].

**Fig. 1:**
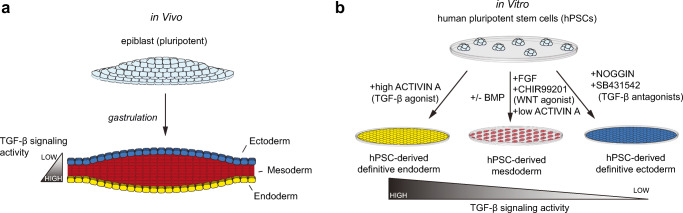
Germ layer specification *in vivo* (**a**) and *in vitro* (**b**). During gastrulation differentiation of the pluripotent epiblast is regulated by TGF-β signaling. Epiblast cells experiencing high levels of TGF-β signaling differentiate to endoderm. Epiblast cells experiencing low levels of TGF-β signaling differentiate to ectoderm (**a**). Stimulation of TGF-β signaling by exogenous ACTIVIN A directs hPSCs to differentiate into endoderm (**b**). In contrast, blocking TGF-β signaling directs hPSCs to differentiate into ectoderm (**b**). Directed differentiation targeting mesoderm stimulates WNT, FGF and intermediate levels of TGF-β signaling with the presence or absence of BMP targeting differentiation toward specific mesoderm sub-populations (**b**)

As hPSCs exit pluripotency and begin to differentiate, the first cell fate decision commits hPSCs to one of the germ layers established early in human development: the endoderm, mesoderm, and ectoderm. In the embryo, the process by which cells make this first decision is referred to as gastrulation. Studies of gastrulation in model organisms have identified key signaling pathways and their requirements for endoderm, mesoderm, and ectoderm formation [48–50]. These studies point to members of the TGF-β signaling superfamily as important signaling molecules that regulate cell fate choice towards a particular germ layer, with NODAL playing a key role in stimulating TGF-β signaling to promote endodermal differentiation in vivo. In contrast, the absence of TGF-β signaling promotes ectodermal cell fate (Fig. 1a). This knowledge has been leveraged in vitro to guide hPSCs through germ layer specification and protocols to generate near-pure populations of endoderm, mesoderm, and ectoderm have been developed [51–55]. These protocols mirror development, by either stimulating TGF-β signaling with ACTIVIN A to target endoderm [51, 54], or by inhibiting TGF-β signaling to target ectoderm [53]. Conditions to induce mesoderm formation stimulate WNT, FGF, and intermediate levels TGF-β signaling, with modulation of BMP signaling altering differentiation to target particular sub-populations of mesoderm [52, 55–57]. Thus, by manipulating TGF*-*β signaling in vitro, hPSCs can be reproducibly differentiated towards a specific germ layer (Fig. 1b). It is important to note that many protocols to target differentiation towards the desired germ layer(s) exist; however, generation of organoids containing cells from multiple germ layers (i.e., epithelial and mesodermal/stromal) requires co-differentiation of both lineages within a culture. This is often achieved by differentiating to one germ layer with less than perfect efficiency (i.e., endoderm), resulting in co-differentiation of other lineages (i.e., mesoderm).

### Establishing regional identity

**Fig. 2:**
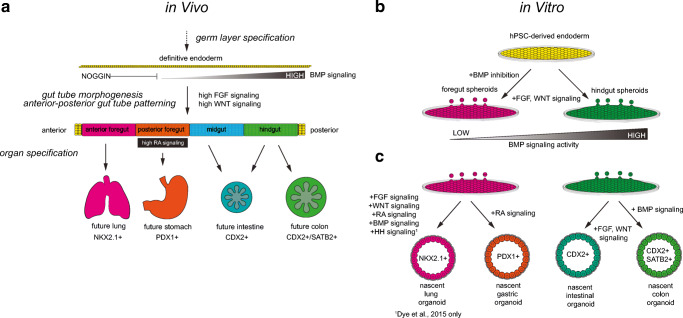
Regional patterning of the endoderm and specification of organ-specific progenitors in vivo (**a**) and in vitro (**b**, **c**). In vivo WNT, FGF, and BMP signaling pattern the gut tube into regional domains along the anterior to posterior axis, with high levels of BMP signaling promoting posterior regional identities. NOGGIN is localized to the anterior region where it represses BMP signaling to specify foregut regional identities. Retinoic acid (RA) signaling further regulates further regionalization of foregut lineages. Regional patterning restricts the developmental trajectory of endoderm towards specific organs. Molecular markers of organ identity are shown below each organ (**a**). hPSC-derived definitive endoderm can be further differentiated to make foregut or hindgut spheroids by manipulating BMP signaling in the presence of WNT and FGF signaling agonists (**b**). To make organoids, hPSC-derived endoderm spheroids are embedded in 3D extracellular matrix and are further differentiated towards organ-specific progenitors. These methods include increasing RA signaling to differentiate gastric progenitors from foregut spheroids and increasing BMP signaling to differentiate hindgut spheroids towards colonic progenitors. When organoid specification is complete, organoids express the same molecular markers as their in vivo counterparts (**c**)

Differentiating hPSCs towards the endoderm lineage is the first step to make organoids representing endodermally derived organs, such as the esophagus, lung, stomach, liver, intestine, or colon. The next step of directed differentiation attempts to recapitulate developmental signaling that further patterns endoderm into specific regional identities—ultimately determining organ identity. In vivo, these regional endoderm identities correspond to their location within the embryo, with identity first being established along the anterior to posterior (head-to-tail) axis. This leads to patterning of the endoderm into an anterior foregut domain, which gives rise to the thyroid, esophagus, trachea and lung, a posterior foregut domain giving rise to the stomach, liver and pancreas, a midgut domain which generates digestive portions of the intestine, and a hindgut domain giving rise to the remaining portions of the intestine including the majority of the colon (Fig. 2a). In relation to other germ layers, regional patterning of the endoderm is akin to patterning of the ectoderm into regions of central nervous, neural crest, and epidermal differentiation.

The response of hPSC-derived endoderm to cell signaling pathways in vitro is similar to that of endoderm undergoing development in vivo with respect to both inducing regional anterior posterior identity and to recapitulating aspects of gut tube morphogenesis. Studies from animal models point to a conserved signaling network involving WNT, FGF, and BMP signaling responsible for patterning regional identity in the vertebrate endoderm [49]. In general, high activity of these signaling pathways promotes posterior identities, while lower activity promotes anterior cell fates. This gradient of signaling pathway activity is established in part by the localization of BMP signaling antagonists, such as NOGGIN to the anterior side of the embryo [58, 59] (Fig. 2a). Accordingly, the presence of NOGGIN or other BMP signaling inhibitors in hPSC-derived endoderm cultures promotes anterior foregut identity, and is an essential step in the generation of foregut-derived organoids such as the lung and stomach [16, 20, 34, 60–67] (Fig. 2b). Alternatively, placing hPSC-derived endoderm into conditions that stimulate WNT and FGF signaling, while permitting endogenous BMP signaling to occur promotes mid-and-hindgut identity and is an important step for the formation of intestinal organoids [68–71] (Fig. 2b).

Remarkably, stimulation of FGF and WNT signaling in vitro is sufficient for both foregut and hindgut patterned hPSC-derived endoderm to engage morphogenic processes akin to the formation of the primitive gut tube in vivo, forming aggregates of epithelial progenitors and mesenchyme precursors that bud up from the culture dish and pinch off to form 3D spheroids [34, 64, 69]. Spheroids are named according to their regional identity, with foregut endoderm cultures giving rise to foregut spheroids, and hindgut endoderm cultures giving rise to hindgut spheroids, which are then embedded into an extracellular matrix such as Matrigel to permit continued expansion and directed differentiation in 3D (Fig. 2b).

### Establishment of organ-specific progenitors

After patterning hPSC-derived endoderm towards a specific region along the anterior posterior axis and transitioning to 3D growth in extracellular matrix, cell fate is further refined to generate progenitors for specific organs, a step that is required for long-term growth.

Methods to induce and maintain tissue-specific progenitors have relied on conserved transcription factors that are known to mark specific progenitor populations, such as NKX2.1 in lung epithelial progenitors and CDX2 in intestinal epithelial progenitors. These markers are useful to screen for conditions that enhance marker expression, signifying a more efficient and targeted differentiation towards a particular fate. This approach has identified Hedgehog, WNT, FGF, Retinoic acid (RA), and TGF-β signaling pathways as pathways that can be stimulated in culture to further differentiate foregut endoderm into lung progenitors [34, 62, 70] (Fig. 2c). Alternatively, extending RA treatment in the absence of simulating other signaling pathways directs foregut endoderm to generate gastric progenitors [64, 72] (Fig. 2c), consistent with the role of RA signaling in regulating progenitor identity in the posterior foregut endoderm in vivo [73, 74]. Likewise, regional identity can be further refined in hindgut spheroids towards sub-regions of the intestine, such as the duodenum or ileum by varying the length of WNT and FGF signaling stimulation [75] or by exogenously stimulating BMP signaling to further posteriorize hindgut endoderm towards a colonic epithelial progenitor identity [71] (Fig. 2c).

In directed differentiation protocols where 3D structures do not form naturally, regionalized endoderm is further differentiated towards specific progenitors in 2D culture which are then purified and embedded as single cells in extracellular matrix to facilitate self-organization and expansion into 3D structures. This approach has been utilized in the generation of various types of lung organoids [20, 63, 66]. To this end, molecular profiling of lung progenitors from fetal tissue has identified useful cell surface markers for the isolation of lung progenitors [63, 76]. Alternatively, other groups have applied genome engineering to create hPSC lines with fluorescent reporters of lung progenitor identity, permitting real-time monitoring of progenitor cell specification and dynamics in culture, as well as their isolation [20, 66]. hPSC lines with reporters of intestinal progenitor identity have also been utilized to follow the dynamics of intestinal progenitor specification in culture, and to purify intestinal progenitors away from mesenchyme precursors to generate epithelial only intestinal organoids [72, 77].

After progenitor cells are specified, nascent organoids are shifted from progenitor-inducing media to progenitor expansion and maintenance media, leading to continued organoid growth that mirrors aspects of organ development in vivo. For example, HIOs under maintenance conditions undergo an initial 2-week period of intestinal progenitor expansion, followed by the emergence of intestinal epithelial cell types, such as enterocytes and Goblet cells and LGR5+ intestinal stem cells [37, 69]. Concurrently, HIOs undergo morphogenic events similar to those occurring during fetal intestinal development, including columnar organization of the epithelium, the emergence of sparse finger-like protrusions into the organoid lumen, reminiscent of a nascent villus [69]. Therefore, HIOs recapitulate aspects of fetal intestine development including cell type organization and morphogenesis as they are maintained in culture.

Methods to generate hPSC-derived HLOs are more varied than those to generate HIOs. This may either reflect a fundamental difference in lung and intestinal progenitor cell specification, or may simply reflect preference for a particular protocol/method. HLOs share many aspects with fetal lung development as they expand in culture. HLOs maintained in conditions of high FGF signaling give rise first to NKX2.1+/SOX2+/SOX9+ epithelial cells, reminiscent of early lung progenitors [34]. Alternatively, if HLOs are put into more complex media stimulating BMP, FGF, RA, and WNT signaling, lung epithelial progenitors undergo extensive branching reminiscent of branching morphogenesis during lung development [65]. Changing media conditions lead to the emergence of proximal airway TP63+ basal cells within the first 25 days of culture, followed by cells expressing markers of the alveolar epithelium, similar to the timing of cell differentiation during fetal lung development in which the specification of airway epithelial precedes the appearance of alveolar cells. Moreover, when cultured under conditions permitting differentiation, HLOs exhibit airway to alveolar organization similar to the human lung epithelium, with cells expressing markers of airway predominantly localized inside HLOs and cells expressing markers of the distal lung predominantly localized externally [16, 65].

Alternative to the methods described above, in which progenitor cells undergo expansion accompanied by stochastic differentiation, HLOs enriched for populations of specific lung epithelial progenitor cell types can also be generated. For example, conditions promoting long-term maintenance of nearly pure populations of lung epithelial progenitors, called bud-tip organoids (BTOs) have been established [16]. Notably, establishment of these conditions relied heavily on the isolations and characterization of progenitors present in the tips of developing fetal lungs [15, 16]. Methods also exist to generate HLOs enriched for more committed lung progenitors such as TP63+ airway basal cells [17, 78] or for alveolar type II cells [66]. Specification and maintenance of TP63+ basal cells in HLOs are achieved by manipulating TGF-β signaling activity, mirroring regulation of basal cell specification by TGF-β signaling in vivo [79]*.* Likewise, differentiation of alveolar type II cells from hPSC-derived lung endoderm is promoted by glucocorticoid and WNT signaling pathways, which have also been implicated in promoting alveolar cell fate specification in murine lung development [80–82]. The existence of multiple types of HLOs, enriched for different lung progenitor cell types which are responsive to signaling pathways that regulate their behavior during development in vivo provide*s* exciting flexibility to model transitions between lung progenitor cell states in vitro using HLOs [67].

### Strategies to increase organoid maturity

A variety of approaches exist to drive maturation of organoids, which typically describe further patterning of organoids into domains of differentiated and progenitor cells, an increased proportion of differentiated cells, and increased functionality in differentiated cells. The simplest approach is to allow time for the organoids to mature; organoids exhibit an inherent capacity to mature and if left undisturbed in culture and typically increase in cellular diversity and continue to emulate features of early fetal organogenesis over time. However, this capacity is limited, as organoids cultured for multiple months still exhibit transcriptional and proteomic profiles consistent with fetal tissue rather than adult tissue. In addition, as organoids increase in size and complexity, issues such as nutrient and oxygen availability arise and can lead to necrosis in the organoid core.

Intriguingly, transplantation of organoids into amenable sites in a mouse host results in maturation far beyond those of organoids cultured in vitro [37, 65, 83–86]. For example, HIOs transplanted under the kidney capsule of immunodeficient mice exhibit enhanced expression of brush border enzymes and increased cellular differentiation and formation of villus and crypt structures reminiscent of functional intestinal epithelium [37, 85]. Likewise, HLOs transplanted in a similar manner form structures resembling the respiratory airway, with increased epithelial cell diversity relative to that observed in vitro [65, 86]. Along with the hallmarks of maturation noted in the above examples, transplanted HIOs and HLOs become highly vascularized by host cells, increase in size beyond the limit observed in vitro, and exhibit increased mesenchymal diversity and organization [37, 65, 85, 86].

Undoubtedly, the in vivo environment presents chemical cues whose organoid maturing effects have yet to be recognized. Thus, there is likely much work to be done on refining culture conditions to promote maturation of hPSC-derived organoids. Indeed, even in the case of adult stem-derived organoids, refinement of the composition of organoid maintenance media greatly increases cellular diversity [87]. In addition, the association of vascularization and increased mesenchymal complexity with organoid maturation has generated interest in the application of co-culture systems to exogenously seed hPSC-derived organoids with endothelial and other mesenchymal cell types. Such systems have been demonstrated to increase maturation in hPSC-derived brain, lung, liver, and kidney organoids [63, 83, 84, 88, 89]. The presence of endogenous endothelial cells has been demonstrated in hPSC-derived kidney organoids, raising the possibility that other hPSC-derived organoid models may possess similar potential for self-vascularization [36, 44, 89, 90]. The above approaches rely on self-assembly of endothelial cells into networks of vasculature, and alternatively approaches to bioengineer complex vascular networks to support organoid growth and maturation are also rapidly advancing [91].

Finally, great interest exists in the development of bioengineered approaches that attempt to recapitulate physical and other forces acting as maturation cues during development that are not easily translated to the tissue culture dish. For instance, mechanical forces such as tension and extracellular matrix stiffness have long been appreciated as important cues for cellular differentiation and behavior [92]. While Matrigel is widely employed as a growth substrate to support 3D growth of organoids, a variety of synthetic hydrogel compositions have been demonstrated to support the growth and the developmental potential of HIOs offering a more defined alternative with tunable properties such as stiffness and composition [93, 94]. In addition, complex growth substrate matrices that endow spatial and temporal control over substrate stiffness and composition are currently being developed [95, 96]. Microfluidic platforms are also being engineered to recapitulate other physical developmental cues such as flow, and to engineer growth factor/morphogen gradients to generate reproducible organoid architectures [97–99].

Heavily influenced by our understanding of forces that guide organ maturation during development, the above approaches offer promising avenues to mature hPSC-derived organoids in vitro. Moreover, recapitulation of organ maturation in the culture dish is by its very essence a reductive approach, promising exciting insights into human development and mechanisms of human disease.

### Current and future applications for organoids

Although much work remains to improve the complexity and reproducibility of hPSC-derived organoid cultures, hPSC-derived organoids have the potential to provide insight into important areas of medical research, including genetic disease, host-microbe interactions, emerging viral infections, and pre-term infant care. For example, HIOs have been applied to model the contribution of the microbiome to fetal development and human disease by infection with commensal or pathogenic microorganisms [100–103]. Intriguingly, infection of HIOs with non-pathogenic *E.coli* leads to enterocyte maturation in HIOs, suggesting microbe colonization can act as a maturing force in hPSC-derived organoids [103]. Feasibility of HIOs for studying common viral infections has also been demonstrated [104]. HLOs have also been applied to model common respiratory infections such a respiratory syncytial virus [65, 105]. Of particular interest, adult stem cell-derived HLOs have been utilized as platforms to assess infectivity of emerging influenza strains [106, 107], suggesting hPSC-derived HLOs could provide a renewable source of respiratory tissue for modeling emerging and common respiratory infections.

In addition, hPSCs are amenable to genome engineering and provide an opportunity to derive iPSC lines from patients carrying disease-associated mutations, making hPSC-derived organoids particularly well suited for modeling genetic disease. iPSC lines derived from patients have been shown to recapitulate pathologic phenotypes in retinal [108], lung [20, 66, 78], kidney [21], intestinal [109], and colonic [110, 111] organoids, among many others. In many of these studies, genome engineering is applied to generate mutation-corrected iPSC lines, which permit the comparison of organoid development between genetic backgrounds that only differ at the site of the mutation under investigation. This strategy of comparing organoids from patient-derived and mutation-corrected iPSCs is an invaluable approach to assessing the contribution of individual risk variants to disease phenotypes.

hPSC-derived organoids are particularly applicable to modeling diseases associated with pre-term birth since they resemble developing fetal tissue [32–34, 36, 37, 84]. Due to the relatively late developmental timing (3rd trimester) at which the lung becomes competent for gas exchange, diseases associated with pre-term birth are especially relevant to HLOs. Therapies to support respiration in pre-term infants include mechanical ventilation, which can induce long-term lung disease known as bronchopulmonary dysplasia [112]; thus, alternative therapies are desperately sought-after. Glucocorticoid steroids have long been recognized for their ability to accelerate surfactant production [113–115], a hallmark of lung maturation. HLOs can be applied to investigate the mechanism by which glucocorticoids promote lung maturation, leading to improved treatment regimens for pregnancies at significant pre-term birth risk. Additionally, HLOs are already an excellent platform to screen for additional molecules accelerating the development and maturation of human lung cells, leading to novel therapies to rescue lung function in pre-term infants.

HIOs also present opportunities for application to pre-term or low birthweight-associated disease. In particular, a major issue for pre-term infants is nutrient delivery. In utero, nutrient delivery occurs through the placenta. In the neo-natal intensive care unit, doctors must choose between delivering nutrients parenterally (i.e., intravenously), a highly invasive procedure leading to numerous complications, or enterally, which has high risk for the development of necrotizing enterocolitis (NEC). NEC is thought to arise due to a confluence of factors including epithelial barrier dysfunction in the immature fetal GI tract, likely mediated by microbial colonization, premature feeding, and associated inflammation [116, 117]. Methods have been developed to assess barrier function in HIOs and to inoculate HIOs with microbes [101–103, 118], presenting opportunities to model fetal intestinal barrier function in different microbial contexts with HIOs. Additionally, feasibility of modeling nutrient uptake has been demonstrated in primary intestinal organoids from mice [119], and the development of analogous methods in hPSC-derived HIOs will permit modeling of human fetal intestine nutrient uptake and the development of therapies that accelerate maturation involved with nutrient uptake function in the fetal intestine.

### Promise and Challenges

hPSC-derived organoids hold great potential for investigating human specific aspects of development and disease. In their current form, hPSC-derived organoids are excellent models of early fetal development, presenting opportunities to model congenital disease and to improve pre-term neonatology. Efforts are currently underway to increase the complexity and maturity of hPSC-derived organoids, with the eventual goal of using hPSC-derived organoids to model all stages of organ development, from fetal, through adulthood, and in aging. Achievement of this goal will have profound impact on human health, but in order to realize this great promise, important challenges will need to be addressed.

A long-standing challenge for the field remains the standardization of hPSCs from different sources and across different laboratories. For the purposes of this review, we referred to pluripotent stem cells derived from embryos (ESCs) and patients (iPSCs) collectively as hPSCs; however, fundamental differences exist between ESCs and iPSCs, including their differentiation and tumorigenic potential. Epigenetic memory of the cell type of origin has been proposed to partially account for differences in differentiation potential among iPSC lines [120, 121], while other studies have argued that genetic background underlies differences between iPSC lines [122]. In addition, multiple methods to generate iPSC cells are employed, some with higher potential to malign the resulting product than others. Because of these potential differences, it is imperative that any significant insight derived from organoids be demonstrated across multiple hPSC lines where possible. To this end, guidelines for defining and identifying clinical grade iPSC lines have been proposed [123], and widespread adoption of such criteria as well as standardization of culture conditions will help to avoid future controversy.

Additionally, because hPSC-derived organoids begin life as pluripotent cells, a unique challenge to application of hPSC-derived organoids is the presence of contaminating cell types in some differentiation protocols. These cells can be either incorrectly specified as the result of sub-optimal differentiation conditions or the result of cells failing to maintain lineage commitment, a result likely reflective of sub-optimal organoid maintenance conditions. As mentioned previously, having sub-pure mixed cultures are advantageous, permitting the co-derivation of mesenchyme and epithelium to generate organoids consisting of multiple germ layers. In other cases, cell types from different organs may be mixed to an extent that makes experimental interpretation meaningless in relation to human physiology. Thus, although there has been much progress made in recent years, particularly with the application of single-cell technologies to refine differentiation protocols, there is still much room for improvement.

Another persistent challenge will be access to tissue necessary to benchmark in vitro*-*derived organoids. While insights from the development of model organisms will undoubtably provide important context, as hPSC-derived organoids become more similar to the in vivo organs they model, uniquely human aspects of organ development and physiology will become apparent. Thus, benchmarking hPSC-derived organoids against both fetal and adult human tissue is a necessary step to ensure the accuracy of organoid systems [17, 33, 40–45]. Although these studies operate under very strict regulatory and ethical guidelines and utilize fetal tissue specimens that would otherwise be discarded, the use of such tissue remains a polarizing issue in the USA and in other parts of the world. In particular, the use of fetal tissue in research has become entangled with complex political and societal issues, such as abortion, requiring careful consideration for how this research may benefit society as a whole. Therefore, it is imperative that scientists counter misinformation, continue to highlight the societal benefit of their research that utilizes both hPSC-derived tissues and where necessary, fetal tissue, and participate in the development of an ethical and regulatory framework that permits the continuation of such research.

It is remarkable how far hPSC-derived organoids have come in the approximately 20 years since the derivation of the first human pluripotent stem cell line [46]: from essential discoveries of how to coax hPSCs to differentiate towards specific germ layers, to identification of signaling pathways that promote the emergence of particular organ progenitors, to the creation of 3D models of organ development and function. As hPSC-derived organoid models continue to increase in complexity and fidelity, we can look forward to an increased understanding of human-specific aspects of development and disease leading to improved human health.
