# Correction to: hPSC-derived organoids: models of human development and disease

**DOI:** 10.1007/s00109-020-02007-5

**Published:** 2020-11-17

**Authors:** Tristan Frum, Jason R. Spence

**Affiliations:** 1 Department of Internal Medicine, Gastroenterology, University of Michigan Medical School, Ann Arbor, MI USA; 2 Department of Cell and Developmental Biology, University of Michigan Medical School, Ann Arbor, MI USA; 3 Department of Biomedical Engineering, University of Michigan College of Engineering, Ann Arbor, MI USA

**Correction to: Journal of Molecular Medicine**

10.1007/s00109-020-01969-w

The article hPSC-derived organoids: models of human development and disease, written by Tristan Frum and Jason R. Spence, was originally published electronically on the publisher’s internet portal on 28 August 2020 without open access. With the author(s)’ decision to opt for Open Choice the copyright of the article changed on 29 October 2020 to © The Author(s) and the article is forthwith distributed under a Creative Commons Attribution 4.0 International License, which permits use, sharing, adaptation, distribution and reproduction in any medium or format, as long as you give appropriate credit to the original author(s) and the source, provide a link to the Creative Commons licence, and indicate if changes were made. The images or other third party material in this article are included in the article’s Creative Commons licence, unless indicated otherwise in a credit line to the material. If material is not included in the article’s Creative Commons licence and your intended use is not permitted by statutory regulation or exceeds the permitted use, you will need to obtain permission directly from the copyright holder. To view a copy of this licence, visit http://creativecommons.org/licenses/by/4.0.

The Original article has been corrected.

